# N-Dimensional Reduction Algorithm for Learning from Demonstration Path Planning

**DOI:** 10.3390/s25072145

**Published:** 2025-03-28

**Authors:** Juliana Manrique-Cordoba, Miguel Ángel de la Casa-Lillo, José María Sabater-Navarro

**Affiliations:** Bioengineering Institute, Miguel Hernandez University of Elche, 03202 Elche, Spain; jmanrique@umh.es (J.M.-C.); mcasa@umh.es (M.Á.d.l.C.-L.)

**Keywords:** learning from demonstration, hidden Markov models, data reduction, Douglas–Peucker algorithm, high-dimensional data encoding

## Abstract

This paper presents an *n*-dimensional reduction algorithm for Learning from Demonstration (LfD) for robotic path planning, addressing the complexity of high-dimensional data. The method extends the Douglas–Peucker algorithm by incorporating velocity and orientation alongside position, enabling more precise trajectory simplification. A magnitude-based normalization process preserves proportional relationships across dimensions, and the reduced dataset is used to train Hidden Markov Models (HMMs), where continuous trajectories are discretized into identifier sequences. The algorithm is evaluated in 2D and 3D environments with datasets combining position and velocity. The results show that incorporating additional dimensions significantly enhances trajectory simplification while preserving key information. Additionally, the study highlights the importance of selecting appropriate encoding parameters to achieve optimal resolution. The HMM-based models generated new trajectories that retained the patterns of the original demonstrations, demonstrating the algorithm’s capacity to generalize learned behaviors for trajectory learning in high-dimensional spaces.

## 1. Introduction

Robotic trajectories are essential for various applications, including task planning, energy efficiency optimization, and performing surgical procedures. A robotic trajectory defines the path, speed, and acceleration of a robot’s end effector over time. These trajectories are typically designed by taking into account variables such as the robot’s kinematics, dynamics, and physical constraints, as well as external factors like obstacles and the working environment [1].

In addition to industrial or service applications, robotic trajectories are also crucial in robotics research and development. Learning from Demonstration (LfD) is a method for teaching robots new skills by providing examples of the desired behavior. In the context of LfD, trajectory learning involves modeling and generalizing the movements demonstrated by a human operator [2]. The primary advantage of this approach is that it eliminates the need for extensive technical knowledge to program robots. The intuitive programming style of LfD has the potential to facilitate robotic applications in both the manufacturing and service sectors (e.g., clinical, office, or home environments). In robotics, LfD is often used for skill acquisition at the trajectory level, which involves modeling the demonstrated set of trajectories and obtaining a generalized representation suitable for reproduction by a learner robot [3]. To capture the stochastic variations of human movements in demonstrations, many researchers have employed probabilistic representations of data obtained from multiple repetitions of the same skill. For instance, the literature reports the use of Hidden Markov Models (HMMs) [2,3,4] and Gaussian Mixture Models (GMMs) [3,5,6] for modeling task demonstrations at the trajectory level. Additionally, probabilistic movement primitives (ProMPs) have been a widely-used approach in robot skill learning to model human motions [7,8]. Among these methods, HMM has been one of the most widely used techniques in the field of robotics LfD for modeling and analyzing human movements due to its robustness to spatiotemporal variations in sequential data [2].

While LfD emphasizes the acquisition and generalization of complex human-like movements for robotic applications, trajectory simplification techniques offer a complementary approach by optimizing the training data for efficient processing and implementation. In LfD, trajectory learning often involves high-dimensional data with stochastic variations inherent to each demonstration, such data can be computationally intensive. Simplification algorithms like Douglas–Peucker [9] or Visvalingam–Whyatt [10] can reduce this complexity by condensing trajectory data without losing essential path characteristics, thereby enhancing the feasibility and responsiveness of LfD-trained robots in practical settings. Integrating simplification techniques with LfD could, therefore, improve both the efficiency and adaptability of robots across various domains, including manufacturing, service, and clinical environments.

The simplification of geometries and trajectories is a fundamental technique in robotics, navigation systems, and computer graphics, aimed at reducing the number of points in paths or contours while preserving their essential shape. This optimization decreases processing time, reduces memory consumption, and accelerates trajectory planning. Among the classic algorithms for this purpose is the Douglas–Peucker algorithm, initially applied to cartographic generalization, which reduces vertices by removing points from a line based on a maximum set distance [9]. Another method is the Visvalingam–Whyatt algorithm, which simplifies by removing points with lower “effective area”, thus preserving the line structure [10]. The sleeve-fitting approach adapts segments to a virtual “sleeve”, discarding minor variations and facilitating tracking applications [11]. The Reumann–Witkam algorithm simplifies trajectories by retaining points within a “band” parallel to the initial segment, making it efficient for straight or slightly curved lines, although it may introduce distortions in complex trajectories [12].

Most recent studies on geometry and trajectory simplification algorithms focus primarily on two-dimensional or, at most, three-dimensional environments. When additional dimensions are considered, they typically rely exclusively on motion-related information [13]. The main applications of these algorithms include cartographic simplification, such as web mapping, where modifications to the Douglas–Peucker algorithm have been proposed to enhance its performance in this domain [14]. Additionally, several methods have been developed to enable online trajectory simplification for real-time location services [15].

Some studies have integrated LfD into robotic trajectory generation using simplified trajectories. For instance, Vakanski et al. [3] employ the Linde–Buzo–Gray (LBG) algorithm [16] to select key points, which are then used in HMMs for trajectory learning. These approaches highlight the potential of combining trajectory simplification with data-driven learning techniques to enhance robotic motion planning.

This work explicitly extends trajectory simplification by incorporating additional parameters beyond position, addressing the challenge of data heterogeneity through magnitude-based normalization. To achieve this, the Douglas–Peucker algorithm was selected and modified for implementation in a multidimensional environment. The choice of this algorithm is based on its computational efficiency, as it employs a recursive approach for key-point selection, making it well-suited for high-dimensional trajectory processing.

Specifically, this study evaluates the impact of speed and/or orientation variations at each trajectory point to determine whether these factors significantly contribute to the simplification process while preserving essential motion characteristics. By normalizing different magnitudes, the proposed approach ensures that all parameters are treated consistently, facilitating a more accurate and robust simplification framework. This enhancement enables the method to maintain the structural integrity of trajectories while optimizing data reduction for subsequent learning models.

The structure of the paper is as follows: Section 2 presents details of the algorithm used. First, the pre-processing of the generated data is presented, including normalization, followed by simplification. A detailed explanation of the proposed *n*-dimensional Douglas–Peucker algorithm is provided. Next, the implementation of the HMM is explained, specifying the generation of the training data and the initial matrices provided to a model devoted to generating new robotic trajectories that meet the requirements of the key points.

The results of the proposed experiments are presented in Section 3, applying the procedure to digitally generated trajectories in two-dimensional and three-dimensional spaces. These experiments are conducted using, on the one hand, only position data and, on the other hand, the combined position–velocity dataset. Finally, the Section 4 outlines the improvements that should be included in order to analyze more complicated approaches.

## 2. Materials and Methods

**Figure 1:**
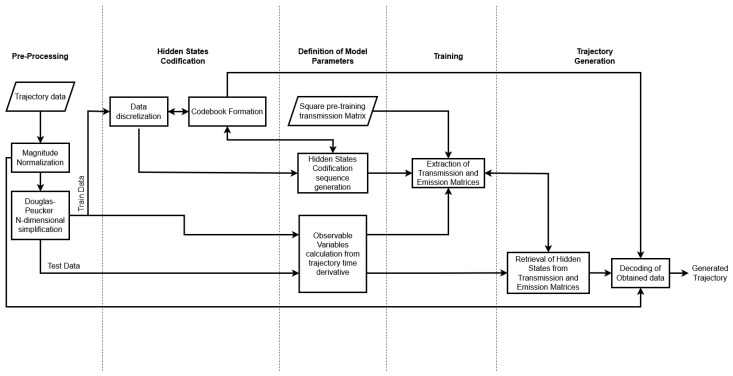
Flowchart of the proposed framework.

Figure 1 outlines the step-by-step methodology for processing data in the study. It begins with trajectory data generation, followed by a normalization by magnitudes to ensure the homogeneous treatment of the data. Next, it undergoes feature extraction through the Douglas–Peucker simplification to identify key parameters. The extracted features are then fed into a machine learning model and trained using hidden Markov models to learn complex patterns from the data. This stage includes defining the transition and emission matrices. Finally, the system outputs the predicted states to be decoded into the proposed trajectory.

### 2.1. The 2D/3D Data Generation

Two-dimensional and three-dimensional trajectories are generated at a constant frequency, with each trajectory repeated at both constant and variable speeds at different points. To create a more extensive dataset suitable for training the learning model, random noise of 5% is added to positions and 3% to velocities.

The trajectories generated in two-dimensional position (X,Y) produce a four-dimensional dataset, consisting of two position components and two velocity components $\left(X,Y,V_{x},V_{y}\right)$. Similarly, the trajectories generated in three-dimensional position (X,Y,Z) yield a six-dimensional dataset, comprising three position components and three velocity components $\left(X,Y,Z,V_{x},V_{y},V_{z}\right)$.

### 2.2. Magnitude Normalization

Given that the information obtained from the trajectory is heterogeneous (e.g., positions, velocities), normalizing each dataset individually (e.g., positions along each axis) leads to a loss of trajectory information. To address this, normalization is carried out based on magnitude groups, preserving the dimensional proportions and their effect on the trajectory when selecting characteristic points.

Before beginning the preprocessing, it is crucial to record the maximum ($max_{i}$) and minimum ($min_{i}$) values of each physical magnitude group. Here, *i* represents the number of groups of similar variables. This procedure ensures that each group of physical magnitudes is normalized with respect to a common range, calculated from the global maximum and minimum values within the group.

**Figure 2:**
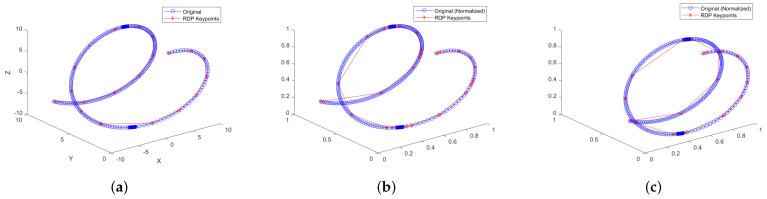
Simplification of a spiral trajectory with variable speed and 6 dimensions $\left[X\;Y\;Z\;V_{x}\;V_{y}\;V_{z}\right]$ using the DP algorithm: (**a**) Three-dimensional visualization without normalization. (**b**) DP Simplification normalized by individual vector plane. (**c**) DP Simplification normalized by unit groups.

The example in Figure 2a shows a spiral trajectory that moves a distance of 20 units along the X-axis (from −10 to 10), while on the Y-axis (from 0 to 10) and the Z-axis (from −5 to 5), it moves only 10 units. When calculating the characteristic points without applying normalization, the result is shown. However, when normalizing by vectors, a similar displacement is observed across all axes, thereby altering the selection of characteristic points (Figure 2b). Therefore, normalization by magnitudes (position, velocity, force, etc.) has been chosen, as shown in Figure 2c, to ensure the correct scaling of the trajectory.

### 2.3. N-Dimensional Douglas–Peucker Reduction Algorithm

In order to generate a parametric trajectories library of different simple maneuvers, an *n*-dimensional trajectory subsampling method is required, allowing us to reduce the order of the learned trajectory while defining key points that can be used as measurement parameters of the trajectory. In addition, a method to represent the obtained key points in the *n*-dimensional space and the time associated with said points is required.

The Douglas–Peucker algorithm implemented starts from a matrix *R* and a tolerance ε; the matrix *R* of size n×m, where *n* is the number of sampled points and *m* is the dimension of every point condenses the information from the trajectory points (*P*) into each row, where each column corresponds to a specific value (e.g., positions, velocities, forces, etc.).

(1) $$R={\left[P_{1},P_{2},P_{3},\cdots ,P_{n}\right]}^{T}$$

(2) $$\begin{array}{ccc} P_{i}=\left[X_{i},Y_{i},Z_{i},V_{i},\cdots ,F_{i}\right] & for & i:1:m \end{array}$$

The algorithm verifies that n>2; if this inequality is not true, *R* is returned as the optimized trajectory (Rdp), otherwise the *n*-dimensional distance *d* that exists between the points $P_{1}$ and $P_{n}$ is obtained as the Euclidean distance:

(3) $$d(n)=\sqrt{{\left(F_{n}-F_{1}\right)}^{2}+\cdots +{\left(Y_{n}-Y_{1}\right)}^{2}+{\left(X_{n}-X_{1}\right)}^{2}}$$

Consecutively, it is intended to calculate the perpendicular distance from the line that connects the points $P_{1}$ and $P_{n}$ to the furthest point of the trajectory. With the distance d(n), an evaluation of the relative positions between the points is conducted. If d(n) is less than the minimum computable distance in the software, in which the calculation is implemented (in Matlab: eps = $2.204\times 10^{-16}$), then the distance to the furthest point will be calculated from point $P_{1}$.

For d(n)<eps:

(4) $$dp(k)=\sqrt{{\left(F_{i}-F_{1}\right)}^{2}+\cdots +{\left(Y_{i}-Y_{1}\right)}^{2}+{\left(X_{i}-X_{1}\right)}^{2}}$$

To evaluate the perpendicular distance dp(k) between a point $P_{i}$ and the line formed by $P_{1}$ and $P_{n}$ in an *n*-dimensional space, it is necessary to construct a square matrix ($A_{d}$) that ensures canonicity. This allows the use of geometric tools to simplify the calculation as follows:

- The first row corresponds to the coordinates of the first point ($P_{1}$) of the line written as $\left[1,X_{1},Y_{1},Z_{1},V_{1},\cdots ,F_{1}\right]$.
- The second row contains the coordinates of the point of interest $P_{i}$, for which the distance is being evaluated, written as $\left[1,X_{i},Y_{i},Z_{i},V_{i},\cdots ,F_{i}\right]$. Where *i* takes values from 2 to n−1.
- The third row includes the coordinates of the second point ($P_{n}$) of the line, written as $\left[1,X_{n},Y_{n},Z_{n},V_{n},\cdots ,F_{n}\right]$.
- Additional rows are added to complete the matrix, with ones on the upper diagonal and zeros on the lower diagonal, ensuring the matrix’s canonicity. The number of additional rows depends on the dimensionality of the space *n*, as the matrix must be square with a size of (n+1)×(n+1).

The volume calculated by $det(A_{d})$ is proportional to the product of the perpendicular distance dp(k) and the length of the base $∥P_{1}P_{n}∥$, therefore:

For d(n)>eps:

(5) $$A_{d}=\left\{\begin{array}{ccccccc} 1 & X_{1} & Y_{1} & Z_{1} & V_{1} & \cdots & F_{1}\\ 1 & X_{i} & Y_{i} & Z_{i} & V_{i} & \cdots & F_{i}\\ 1 & X_{n} & Y_{n} & Z_{n} & V_{n} & \cdots & F_{k}\\ 0 & 1 & 1 & 1 & 1 & \cdots & 1\\ 0 & 0 & 1 & 1 & 1 & \cdots & 1\\ \vdots & \vdots & \vdots & \vdots & \vdots & \ddots & \vdots \\ 0 & 0 & 0 & 0 & 0 & \cdots & 1 \end{array}\right\}$$

(6) $$dp(k)=\frac{\left|det(A_{d})\right|}{∥P_{1}P_{n}∥}$$

Thus, to find the furthest point in the trajectory, a loop is executed from $P_{2}$ to $P_{n-1}$, storing the perpendicular distance between the line joining $P_{1}$ and $P_{n}$ to each point in the trajectory dp(k). Where *k* is the number of evaluated points through the trajectory.

The vector dp stores the distances of the segment of interest of the trajectory to point *k*, and the distance with the greatest value is selected from it; assuming that the furthest point is $P_{i}$, the algorithm evaluates if the distance of this point is greater than the tolerance ε defined for this trajectory. If the distance is greater than the tolerance, a recursive call to the algorithm is made with two sub-trajectories, the first from $P_{1}$ to $P_{i}$ and the second from $P_{i}$ to $P_{n}$, from which the reduced trajectory is built; on the other hand, if the maximum distance dp is less than the tolerance then the reduced path (Rdp) will become [$P_{1}$,$P_{n}$].

To illustrate the selection of key points, the *n*-dimensional Douglas–Peucker algorithm is applied to a simple trajectory in a two-dimensional space with variable velocity. In cases where few key points are obtained, there is a risk that significant features of the trajectory may be lost during the simplification process. In this particular case, the number of points is directly influenced by the tolerance specified in the simplification algorithm.

**Figure 3:**
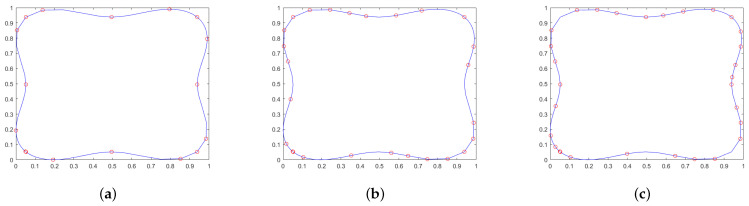
Example of key point selection using the *n*-dimensional Douglas–Peucker reduction algorithm. All figures display the original trajectory in blue, while the red points represent the key points selected for each case. (**a**) Simplification using only normalized position information. (**b**) Simplification using only normalized velocity information. (**c**) Simplification using normalized information of both position and velocity.

In Figure 3a, the selection of points is shown when using only the *X* and *Y* coordinates of each trajectory point, resulting in 17 key points. This is compared to Figure 3b, where velocity information for each axis is included, yielding a total of 26 key points. Finally, the algorithm’s performance is analyzed using normalized position and velocity information, as shown in Figure 3c, where 30 key points are obtained. All of the aforementioned simplifications were performed using the same tolerance, yielding different results depending on the dataset used.

### 2.4. Learning from Demonstration Model

**Figure 4:**
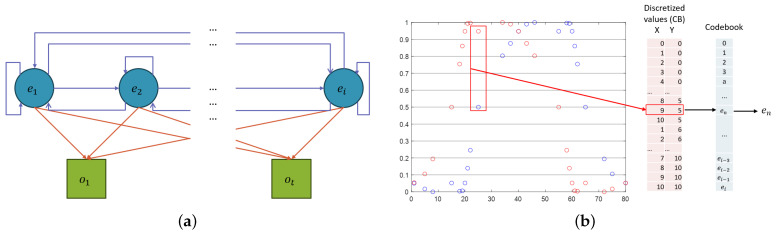
(**a**) Graphical representation of HMM; (**b**) 2D Codebook formation representation.

In this article, a discrete HMM was implemented, making it necessary to map the continuous trajectories into a sequence of discrete values. A graphical representation of an HMM is shown in Figure 4a, where hidden states are denoted with $e_{i}$ and the observable variables are denoted by $o_{t}$.

Once the normalized key points of each trajectory are defined, each dimension is discretized according to the desired resolution, and each value on every dimension is noted as a basic action (q). It is worth noting that dividing the information into more groups leads to greater precision but incurs higher computational cost.

#### 2.4.1. Codebook Formation

Due to the data (Rdp) being normalized by groups within each dimension, according to Section 2.2, with values ranging between 0 and 1, the data are now interpolated to match a number of clusters (CB(i)) into which they will be divided (e.g., 20 groups for positions and 10 groups for velocities), where *i* indicates the number of groups of homogeneous variables in the dataset. The value of (CB(i)) is directly related to the desired resolution for discretizing each variable. The signal is then discretized to the nearest lower integer.

(7) q=[Rdp×CB(i)]

where *q* represents the discretized value of the signal for each dimension. In order to assign a code to the discretized values, a matrix is generated. This matrix has a number of columns equal to the number of data vectors and a number of rows equivalent to the total possible combinations of the discretized values defined by (CB(i)) for each column.

The combination of different discretized values of *q* is assigned to a hidden state code (e) from the discretized values matrix corresponding to an integer of the row position of this specific combination in the codebook, being $e_{i}=\left[q_{1},q_{2},q_{3},\cdots ,q_{n}\right]$.

Figure 4b illustrates the formation of the codebook for hidden states based on the discretization of the Cartesian position variables in 2D. It shows that a respective code is assigned to each possible combination, allowing the corresponding sequence to be generated.

If the signal has *n* dimensions and *p* points, there will be n×p basic actions and a sequence with a total of *p* elements. The codebook will then condense all possible combinations (m). The hidden states will thus be the coded sequence of the combination of corresponding basic actions.

(8) $$m=\prod_{i=1}^{max(CB)}CB(i)$$

Similarly, the observable variables correspond to the time progression at which each point is recorded. Based on the assumption that the training trajectories are performed within a similar timeframe, the time value is normalized and discretized and segmented into $N_{t}$ groups to be input into the HMM.

#### 2.4.2. Pre-Training Transmission Matrix

To prevent zero probability estimations for transitions with very low probabilities that might not be represented in the training sequence, a square pseudo-transition matrix $\left(A_{ps}\right)$ is provided to the training process, indicating that all states initially have an equal probability of transitioning to another state, where $a_{ps(i,i)}=1/m$, with *m* representing the number of possible hidden states defined by the codebook.

#### 2.4.3. HMM Implementation

The transition (A) matrix, size m×m, and the emission (B) matrix, size $m\times N_{t}$, where $N_{t}$ represents the maximum value that the observable variables can take, which in this case, corresponds to the maximum value used to discretize the execution time of the trajectory.

The calculation of matrices *A* and *B* is implemented in MATLAB 2024b through the function *hmmestimate*. This function calculates maximum likelihood estimates of transition and emission probabilities from a sequence of observable variables along with their corresponding hidden states. A more detailed explanation of the HMM training function can be found in [17].

The most likely path through the specified HMM is calculated using the transition matrix *A* and the emission matrix *B*. In this context:

- $A_{\left(i,j\right)}$ represents the probability of transitioning from state *i* to state *j*.
- $B_{\left(i,k\right)}$ represents the probability of emitting symbol *k* from state *i*.

The Viterbi algorithm leverages these matrices to identify the sequence of states that maximizes the likelihood of the observed emissions given the model parameters.

This algorithm is implemented using the *hmmviterbi* function in MATLAB. This function initializes the model in state 1 at step 0, prior to the first emission. The *hmmviterbi* function computes the most likely path based on the assumption that the model starts in state 1.

#### 2.4.4. De-Codification

Before starting the data preprocessing, the maximum ($max_{i}$) and minimum ($min_{i}$) values for each dataset were recorded. This step is crucial since the data generated by the learning from demonstration algorithm are discretized within a specific range defined by ${CB}_{i}$. To map these discretized data back to their original range, as defined by $min_{i}$ and $max_{i}$, a scaling equation is applied:

(9) $$p_{real}=min_{i}+\frac{\left(p_{discrete}+0.5\right)*\left(max_{i}-min_{i}\right)}{CB}$$

where:

- $p_{real}$: Rescaled value in the original range.
- $p_{discrete}$: Discrete value generated by the model.
- CB: Total number of levels in the discretized range.

This process enables the conversion of discretized data back into their original physical scale, preserving consistency with the initial magnitudes of the variables.

## 3. Results and Discussion

To evaluate this proposal, two types of experiments were conducted. First, the simplification and learning of trajectories in a two-dimensional environment were performed, where data correspond to position only and the complete position–velocity set were provided. The same process was then repeated for a trajectory in a three-dimensional environment.

Initially, the trajectories, both two-dimensional and three-dimensional, are generated digitally. To create the training dataset, a set of 100 new trajectories is synthesized by adding 10% random noise to the original trajectory. Once the HMM is trained, a new trajectory is generated with lower noise (5%), simulating the “ideal” execution of the exercise. From this trajectory, time-series data are extracted to encode the observable variables, which are then input into the model to obtain the corresponding hidden states. These hidden states identify the key points of the new trajectory.

### 3.1. Two-Dimensional Environment Trajectories

**Figure 5:**
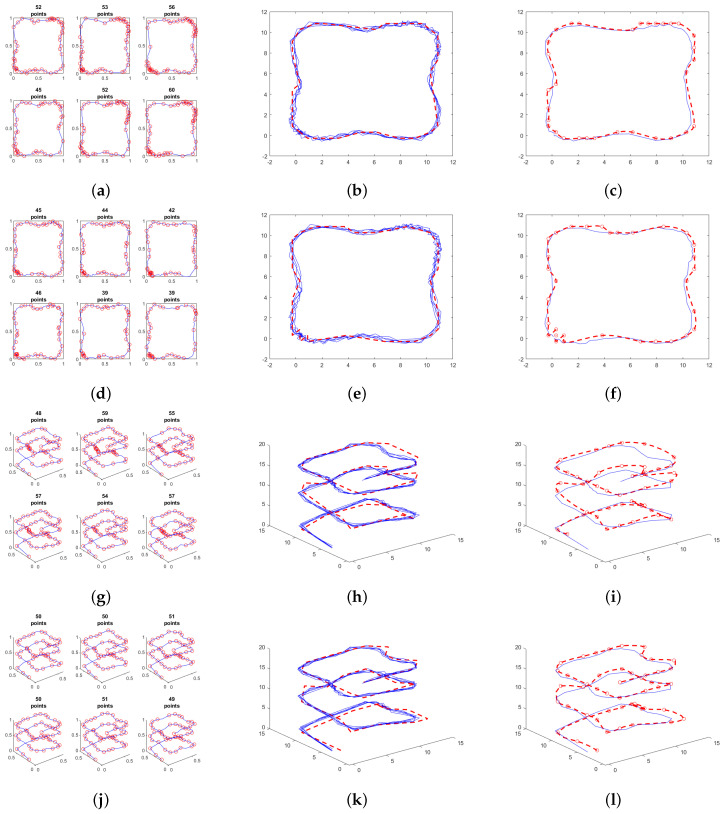
Square trajectory performed in a two-dimensional (**a**–**f**) and three-dimensional (**g**–**l**) space. (**a**) Simplification using only 2D position data. (**b**) Sample of trajectories from the training set (blue), and trajectory generated by the HMM (red) using only 2D position data. (**c**) Original HMM test trajectory (blue). Fitted trajectory (red) obtained from the HMM trained using only 2D position data. (**d**) Simplification using the 4D position–velocity dataset. (**e**) Sample of trajectories from the training set (blue), and trajectory generated by the HMM (red) using the 4D position–velocity dataset. (**f**) Original HMM test trajectory (blue). Fitted trajectory (red) obtained from the HMM trained using the 4D position–velocity dataset. (**g**) Simplification using only 3D position data. (**h**) Sample of trajectories from the training set (blue), and trajectory generated by the HMM (red) using only 3D position data. (**i**) Original HMM test trajectory (blue). Fitted trajectory (red) obtained from the HMM trained using only 3D position data. (**j**) Simplification using the 6D position–velocity dataset. (**k**) Sample of trajectories from the training set (blue), and trajectory generated by the HMM (red) using the 6D position–velocity dataset. (**l**) Original HMM test trajectory (blue). Fitted trajectory (red) obtained from the HMM trained using the 6D position–velocity dataset.

Figure 5a–f illustrates the performance of the simplification algorithm when applied to a trajectory in a two-dimensional space. The learning process was conducted on both a two-dimensional dataset, using the coordinate values (X,Y), and a four-dimensional dataset, incorporating both position coordinates and their corresponding velocities in each axis $\left(X,Y,V_{x},V_{y}\right)$. In both cases, the original trajectory consists of 80 points.

For the two-dimensional simplification, the resulting sets of key points range from as few as 45 points to 60 points. In contrast, the four-dimensional simplification yields sets ranging from 39 to 46 points. A random sample of six trajectories from the training set was selected to be displayed, with each trajectory potentially affected differently by the applied noise.

For the two-dimensional dataset corresponding to spatial coordinates, each vector’s values are discretized into 20 CB groups, deemed sufficient to achieve a satisfactory trajectory resolution. On the other hand, for the four-dimensional dataset, the position vectors are divided into 20 CB groups and the velocity vectors into 10 CB groups, assigning higher resolution to the position data compared to the velocity data.

Figure 5b,e display sample trajectories from the training dataset in blue, compared to the trajectory generated by the learning algorithm in red.

Since the hidden states of the generated trajectory are obtained from the HMM using the observable variables of an “ideal” trajectory, consider an “ideal” trajectory as one generated with a noise level lower than that of the trajectories used during the training of the model. An evaluation is conducted to compare the encoded hidden states of the ideal trajectory with those produced by the HMM. Figure 5c,f illustrate the differences between the original trajectory (blue), from which the observable variables are extracted for input into the HMM, and the fitted trajectory generated (red) from the hidden states obtained from the HMM trained using the 2D position and 4D position–velocity dataset, respectively. In both cases, the new trajectory closely resembles the original trajectory; however, variations indicate that the HMM is not simply replicating the trajectory. Instead, the training data have influenced the HMM’s output, contributing to a new generated response.

### 3.2. Three-Dimensional Environment Trajectories

The results obtained for trajectories in a three-dimensional space are shown in Figure 5g–l. The learning process was executed using a three-dimensional training dataset with only the trajectory coordinates (X,Y,Z) (Figure 5g–i) and a six-dimensional dataset that included position and velocity data $\left(X,Y,Z,V_{x},V_{y},V_{z}\right)$ (Figure 5j–l). In this example, the original training trajectory consists of a square displaced along the *Z*-axis, forming a spiral composed of 71 points with variable velocity.

Figure 5g,j show a sample of the training data that has been simplified using the modified Douglas–Peucker algorithm in 3 dimensions and 6 dimensions, respectively. The simplifications in both cases were performed under the same tolerance. For the 3D case, the sample contains between 48 and 57 points, while for the 6D case, the sample contains between 49 and 51 points.

It can be assumed that in the case where only position data are used, the simplification is directly affected by noise in the signal, as the selection of characteristic points depends solely on the distance potentially influenced by noise between points. On the other hand, when velocity is added to the dataset, the weight of this additional variable impacts the homogeneity of point selection, considering that all trajectories originate from the same base trajectory.

As in the case of three-dimensional data, two graphs are obtained (Figure 5h,k), corresponding to a sample from the training set in blue, compared with the trajectory traced by the points obtained from the HMM in red. The latter closely resembles the traced trajectories; however, for both the 3D and 6D cases, a displacement is observed at the upper part of the HMM trajectory in the values relative to the training trajectories. This is possibly due to the resolution of the encoding groups. The requirement for a significantly larger matrix, resulting from the exponential increase in hidden states when including more dimensions, led to minimizing the number of groups of CB into which the codebook is divided. This reduction could potentially alter the results obtained.

Finally, Figure 5i,l illustrate the difference between the trajectory from which the observable variables were used to feed the HMM and the fitted curve obtained from the points corresponding to the hidden states of the HMM. These figures highlight the similarity between the curves, without one being a replica of the other, as the red trajectory has been influenced by the training data.

### 3.3. Influence of Clustering Resolution

Three two-dimensional models are trained according to the desired resolution by assigning a different number of CB groups to each model. The same test sequence of observable variables is provided to all models to derive the corresponding hidden states that trace a new trajectory.

**Figure 6:**
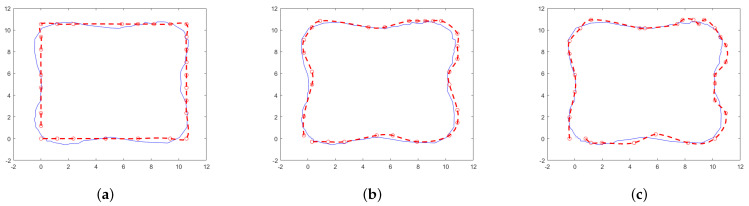
Example of the effect on resolution caused by selecting a different number of CB groups for the discretization of hidden states applied to a two-dimensional dataset: (**a**) 10 CB groups, (**b**) 20 CB groups, (**c**) 30 CB groups.

Figure 6 shows the results obtained for 10, 20, and 30 CB groups. A significant visual difference can be observed between Figure 6a and Figure 6b,c. The resolution with 10 CB groups is considerably lower. The difference between this trajectory and the one generated with 20 CB groups is 25.76%, whereas the difference between the trajectories with 20 and 30 CB groups is 11.89%.

It is important to highlight the impact of coding group selection on the computational cost of model implementation, given the exponential growth of the matrices defining the HMM. For instance, with 10 CB groups, the codebook consists of 100 elements, and the transition matrix contains 100×100 elements. In contrast, 30 CB groups result in a codebook of 900 elements and a transition matrix with 900×900 elements.

## 4. Conclusions

This article presents an alternative approach for selecting characteristic points from a dense dataset, aiming to reduce data volume while minimizing information loss. The proposed method considers the *n* dimensions of the dataset, accounting for potential correlations between them. As a result, an enhanced version of the Douglas–Peucker algorithm is introduced, incorporating the ability to assign corresponding weights to variables beyond spatial coordinates. This modification directly influences the final selection of simplified data points, allowing for more flexible and informative trajectory representations.

The primary focus of this characteristic point selection is to enable its use in training LfD models. Specifically, this study demonstrates its application in learning trajectories using 2D/3D position datasets and 4D/6D position–velocity datasets, utilizing HMMs. The results highlight that incorporating additional dimensions into the selection of key points for trajectory simplification can significantly impact the quality of the preserved information, providing a broader perspective on the final values chosen.

Previous studies have explored the simplification of datasets for various applications, primarily in navigation and localization systems [13,14,15]. These approaches typically focus on 2D and 3D spaces, whereas the algorithm presented in this work is designed specifically for pre-processing data intended for LfD models. A key aspect examined in this study is how trajectory simplification affects the performance of the trained model.

In multidimensional systems, previous research [13] has emphasized the challenge of introduced errors due to data heterogeneity during simplification. This issue is addressed in the present study by applying magnitude-based normalization, which allows data to be treated as a unified set. Additionally, after trajectory simplification and before training the learning models, careful selection of coding group divisions is essential, as it directly affects the resolution of the resulting trajectories. A small number of divisions leads to low resolution, while a larger number improves resolution but may increase computational costs.

Regarding the learning model selected in this study, the literature review highlights the extensive implementation of HMMs as a widely used tool for modeling human motion in free-space environments [4]. HMMs have been particularly applied in humanoid robotics for path planning and obstacle avoidance [2]. This study focuses on employing HMMs as a trajectory planning method based on imitation learning. The results demonstrate that the trajectories generated by the HMM include point sequences that are entirely different from those in the test dataset. This confirms that the model does not merely replicate the training data but instead generates a new trajectory that is fully influenced by the *n*-dimensional simplified training dataset. Consequently, this approach enhances trajectory adaptability while preserving key motion characteristics extracted from human demonstrations.

The extension of this work will focus on applying the proposed algorithm to trajectories captured in an experimental environment, performed by different users and targeted toward specific applications. Additionally, future work will prioritize carrying out a proof-of-concept study and evaluating the performance of the presented algorithm against the existing proposals in the literature.

## 5. Patents

The results presented in this article are documented and protected under the patent with File No. EP25382177 filed on 28 February 2025.

## Data Availability

The data presented in this study are available on request from the corresponding author.
